# Relevance of Diabetic Retinopathy with AGEs and Carotenoid Levels Assessed by Skin Sensors

**DOI:** 10.3390/antiox11071370

**Published:** 2022-07-14

**Authors:** Junichi Sasaki, Yuji Takayanagi, Yoichi Kadoh, Masaki Tanito

**Affiliations:** Department of Ophthalmology, Shimane University Faculty of Medicine, Izumo 693-8501, Japan; m151047@med.shimane-u.ac.jp (J.S.); y.takayanagi1008@med.shimane-u.ac.jp (Y.T.); m171205@med.shimane-u.ac.jp (Y.K.)

**Keywords:** AGEs sensor, skin autofluorescence, Veggie Meter, pressure-mediated reflection spectroscopy

## Abstract

Advanced glycation end products (AGEs) and carotenoids, the major prooxidants and antioxidants in vivo, respectively, are thought to be associated with diabetes mellitus (DM). To estimate AGEs and carotenoid levels simultaneously in patients with DM, we used noninvasive fingertip skin sensors. The study population included 249 eyes of 249 Japanese subjects (130 men, 119 women; mean age ± standard deviation, 69.9 ± 12.0 years). Ninety-three patients had DM, which included diabetic retinopathy (DR) (*n* = 44) and no DR (NDR) (*n* = 49), and 156 controls. Compared to the controls (0.44 ± 0.07 arbitrary unit (A.U.)), the AGEs scores were significantly higher in DM (0.47 ± 0.09, *p* = 0.029) and DR (0.49 ± 0.08, *p* = 0.0006) patients; no difference was seen between NDR (0.45 ± 0.09, *p* = 0.83) and controls. Multivariate analyses indicated that a higher AGEs level is a risk factor for DR (*r* = 0.030, *p* = 0.0025). However, the carotenoid scores did not differ in any comparisons between the controls (327.7 ± 137.0 O.D.) and patients with DM (324.7 ± 126.4, *p* = 0.86), NDR (320.4 ± 123.6, *p* = 0.93), or DR (329.4 ± 130.8, *p* = 0.93). The carotenoid scores correlated negatively with the AGEs scores (*r* = −0.21, *p* = 0.0007), and reflected the Veggie intake score (*p* < 0.0001). In patients with DM, estimations of AGEs and carotenoid levels using skin sensors can be useful for assessing their risk of DR and vegetable intake, respectively.

## 1. Introduction

Diabetes mellitus (DM), a chronic disease characterized by hyperglycemia [1], is associated with an increased risk of microvascular and macrovascular complications [2]. The former include diabetic retinopathy (DR), diabetic kidney disease, and diabetic neuropathy [1], which are the three major complications of DM. DR can cause devastating visual loss [3], and is the leading cause of blindness in middle-aged people [4].

Previous studies have reported possible associations between DM and levels of advanced glycation end products (AGEs) and carotenoids. Elevated blood glucose levels were associated with the accumulation of AGEs [5], suggesting the involvement of AGEs in DM and diabetes complications [6]. In DM, AGEs can alter the function of intracellular proteins such as antioxidant enzymes, increase vascular stiffness by inducing collagen crosslinking, and activate inflammatory signaling pathways by interacting with receptors for AGEs [7,8,9]. On the other hand, higher levels of carotenoids in the blood were associated with a lower risk of developing DM [10]; especially, higher levels of provitamin A (e.g., α-, β-, and γ-carotenes) and β-cryptoxanthin among carotenoids were reported to be correlated with a lower risk of developing DM [11]. However, few studies have simultaneously estimated AGEs and carotenoid levels in patients with DM. The AGEs score estimated by skin autofluorescence (sAF) was correlated positively with the levels of nonfluorescent and fluorescent AGEs in serum [9,12,13]. Because the distributions of fluorescent and nonfluorescent AGEs were similar, sAF reflected the total amount of AGEs accumulation in vivo [8,9]. In addition, the score obtained by the pressure-mediated reflection spectroscopy (RS) method, referred to as the Veggie score, correlated positively with the serum carotenoid levels [14]. The RS method is performed in the 350 to 850 nm wavelength range to include the carotenoid absorption wavelength peak at 480 nm [9]. Thus, these skin sensor-based measurements enable us to determine the in vivo levels of AGEs and carotenoids easily and noninvasively.

In the current study, we estimated the AGEs and carotenoid levels, the major prooxidant and antioxidant molecules in the human body, respectively, using skin sensors in patients with DM, and assessed the roles of these parameters in DM/DR. We also assessed the correlation between AGEs and carotenoid levels.

## 2. Materials and Methods

### 2.1. Subjects

The current study adhered to the tenets of the Declaration of Helsinki. This study was retrospective, and conducted at one institution; the institutional review board of Shimane University Hospital (No. 20200228-2; date of approval, 21 June 2021) approved the study protocol. We reviewed the medical records of the outpatients from 21 November 2019 to 25 May 2021, and selected the patients whose AGEs scores, Veggie scores, and other physical examination data were available. Eyes were excluded if they had retinal lesions, except for DR or glaucoma patients, other than neovascular glaucoma. As a result, a total of 249 eyes of 249 Japanese subjects (130 men, 119 women; mean age ± standard deviation (SD), 69.9 ± 12.0 years) were included. The diagnoses of DM and DR were based on the medical records described by the physicians. Ninety-three patients had DM, of whom 49 had no DR (NDR) and 44 had DR. Among the patients with DM, the eye with the worse DR stage was included. If both eyes had the same DR stage, the eye with worse visual acuity under correction with glasses [i.e., best-corrected visual acuity (BCVA)] was included. If both eyes had the same DR stage and BCVA, the right eye was included. The control subjects were 20 years and older, had no ocular lesions other than age-related cataracts, and the highest intraocular pressure (IOP) (i.e., static pressure inside the eyeball) did not exceed 20 mmHg. Among the control subjects, the eye with better BCVA was included. If both eyes had the same BCVA, the right eye was included. The forced-choice scale with a 4-point rating system was used to estimate the amount of vegetable intake, in which a vegetable intake score of 0 indicated no or rare intake, (1) sometimes/small amount, (2) frequent/sufficient amount, and (3) very frequent/high intake.

### 2.2. Measurement of AGEs in the Fingertip Skin

The AGEs were estimated by measuring the sAF levels, the value of which was obtained using the AGEs sensor (Air Water Biodesign Inc., Kobe, Japan). The measurement was performed using the middle finger of the nondominant hand in which the least skin melanin is present [15]. During the measurement, the fingertip was mildly compressed at the distal portion of the distal interphalangeal joint, which is the suitable region to avoid the nonspecific sAF [13]. The excitation wavelength (365 nm) and emission wavelength (440 nm) were used to obtain the sAF values. The sAF scores were expressed in arbitrary units (A.U.). These measurements were performed two or three times, and the average score was used for statistical analysis. Our pilot study shows that the coefficient of variation and intraclass correlation coefficient (Cronbach’s α) of three repeated AGEs measurements were calculated to be 6.7 ± 7.3% and 0.938, respectively.

### 2.3. Measurement of Carotenoids in the Fingertip Skin

We measured the skin carotenoids by pressure-mediated RS (Veggie Meter^®^, Longevity Link Corporation, Salt Lake City, UT, USA); this score is referred to as Veggie scores. With this method, the influence of blood perfusion was eliminated by the pressure applied to the fingertip, and therefore, skin carotenoid levels were measurable with little influence of melanin pigment [14]. Veggie scores were previously reported to be correlated positively with serum carotenoid levels [14]; thus, we estimated the carotenoid level via the patents’ Veggie score. The measurement was performed using the middle finger of the nondominant hand. White light-emitting diodes (350–850 nm) were used as the light source of RS. The Veggie scores are expressed in optical density (O.D.). These measurements were carried out two or three times, and the average score was used for statistical analysis.

### 2.4. Statistical Analysis

For group comparisons between DM and controls, we calculated the differences in the continuous data using the unpaired *t*-test. The continuous data included age, mean blood pressure, pulse rate (PR), body mass index (BMI), BCVA, IOP, AGEs score, and Veggie score. The decimal BCVA was converted to the logarithm of the minimum angle of resolution (logMAR), and counting fingers, hand motions, light perception, and no light perception were regarded as decimal VAs of 0.0025, 0.002, 0.0016, and 0.0013, respectively, for statistical analysis. We also calculated the differences in the categorical data using Fisher’s exact probability test. The categorical data included sex, current smoking status, lens status, and the vegetable intake score. For group comparisons among NDR, DR, and controls, one-way analysis of variance (ANOVA) followed by post hoc unpaired *t*-tests for continuous data, and G-tests followed by the post hoc Fisher’s exact probability test for categorical data, were performed. The *p*-values of 0.0167 and 0.0033 for the unpaired *t*-tests and Fisher’s exact probability test were considered significant at 5% and 1%, respectively, to correct for multigroup comparisons, which were based on the Bonferroni correction. Multivariate logistic regression analysis was performed to assess the risk factors for DM. To avoid confounding effects between the Veggie score and vegetable intake score, the vegetable intake score was excluded from the multivariate logistic regression analysis. To explore the associations among AGEs and Veggie scores and other parameters, linear regression analyses with Pearson’s correlation coefficient for continuous variables, and unpaired *t*-tests for categorical variables, were performed; multiple regression analyses also were performed. The AGEs and Veggie scores were compared among the vegetable intake score groups (0, 1, 2, or 3) using ANOVA followed by post hoc unpaired *t*-tests. The *p*-values of 0.0083 and 0.0016 were considered significant at 5% and 1%, respectively. Finally, linear regression analysis confirmed the relationship between the AGEs scores and Veggie scores. All statistical analyses were calculated using the JMP Pro statistical software version 16.1.0 (SAS Institute, Inc., Cary, NC, USA). The dataset underlying this manuscript is seen in Table S1.

## 3. Results

Table 1 shows the demographic subject data. Sex, BMI, lens status, BCVA, and AGEs scores differed significantly between the DM and control groups. In addition to these parameters, the mean ages differed significantly among the NDR, DR, and the control groups. The AGEs score was significantly higher in both the DM and DR groups than the controls, but did not differ between the NDR and control groups. The Veggie and vegetable intake scores did not differ significantly between the DM and control groups, or among the NDR, DR, and control groups.

We assessed the risk factors for DM (Table 2) and DR (Table 3) using multivariate logistic regression analysis. In Table 2, higher BMI (odds ratio (OR) = 1.12, *p* = 0.018), pseudophakia (i.e., eyes implanted with intraocular lens by previous cataract surgery) (OR = 6.18, *p* = 0.0008), and worse BCVA (OR = 22.6, *p* < 0.0001) were associated with DM, and female gender (OR = 0.22, *p* = 0.0001) was inversely associated with DM. In Table 3, pseudophakia (OR = 5.25, *p* = 0.0047), worse BCVA (OR = 2.61, *p* = 0.016), higher AGEs scores (OR = 27,659, *p* = 0.0016), and higher Veggie scores (OR = 1.00, *p* = 0.040) were associated with DR, and age (OR = 0.94, *p* = 0.0006) and male gender (OR = 0.31, *p* = 0.013) were inversely associated with DR.

**Figure 1:**
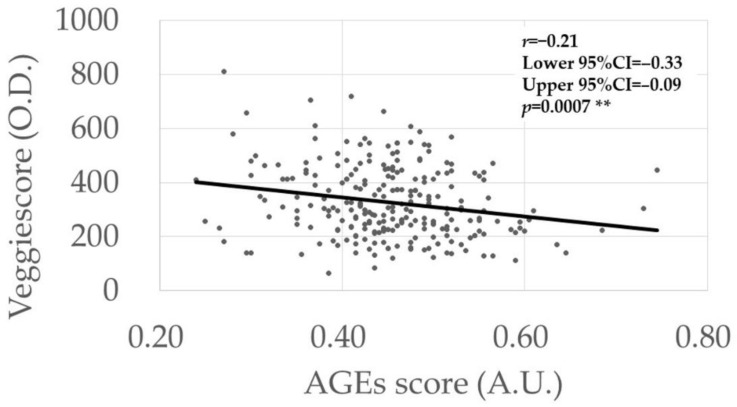
Association between AGEs score and Veggie scores. The *p*-value was calculated via linear regression analysis. ** Significance level at 1% (*p* < 0.01).

The possible associations between the AGEs scores (Table 4) and Veggie scores (Table 5) and various continuous parameters were analyzed using univariate analysis. The AGEs score was correlated negatively with the Veggie score (*r* = −0.21, *p* = 0.0007) (Figure 1), while no correlations were found with the other parameters (Table 4). However, a higher Veggie score was correlated with older age (*r* = 0.13, *p* = 0.049) and better BCVA (*r* = −0.15, *p* = 0.020) (Table 5).

The possible associations between the AGEs scores (Table 6) and Veggie scores (Table 7) and various categorical parameters were analyzed by univariate analysis. In Table 6, the AGEs score was lower in the subjects that were current smokers compared with nonsmokers (*p* = 0.0010). In Table 7, the Veggie score was higher in women (*p* = 0.0018), nonsmokers (*p* = 0.0029), and those with pseudophakia (*p* = 0.014), respectively, that in men, smokers, and phakic groups.

The AGEs and Veggie scores were compared among groups stratified by the vegetable intake scores (Table 8). The AGEs scores did not differ among the four vegetable intake groups, while the Veggie score was significantly higher in vegetable intake group 3 than in the groups 0, 1, and 2, and the group with score 2 was higher than the group with score 1.

Finally, the determinants of the AGEs levels (Table 9) and Veggie scores (Table 10) were assessed by multiple regression analyses. In Table 9, nonsmoking status (*r* = 0.028, *p* = 0.0006), DR (*r* = 0.030, *p* = 0.0025), and lower Veggie scores (*r* = −0.000, *p* = 0.0001) indicated higher AGEs scores. In Table 10, women (*r* = 21.3, *p* = 0.023), nonsmoking status (*r* = 47.3, *p* = 0.0007), better BCVA (*r* = −46.5, *p* = 0.027), and lower AGEs scores (*r* = −479.5, *p* = 0.0001) indicated higher Veggie scores.

## 4. Discussion

In both the univariate and multivariate analyses (Table 1 and Table 9), higher AGEs levels were detected in the comparisons between the control and DM groups and between the control and DR groups, but not between the control and NDR groups. In our previous study, which analyzed the independent dataset, a positive association was found between AGEs level and DR stage progression [7]. Collectively, the current results suggest the roles of systemic AGEs accumulation in the development and progression of DR in patients with DM. However, no differences were seen in the Veggie scores in either disease group comparisons by both univariate and multivariate analyses (Table 1 and Table 10), although an association with the vegetable intake score was detected (Table 8). A negative correlation between the AGEs and Veggie scores was detected in our dataset (Table 4 and Table 5, Figure 1). Simultaneous assessment of AGEs and carotenoid levels in patients with DM using fingertip sensors is unique in the literature.

In addition to the higher AGEs level in DR compared with controls, AGEs indicated a risk for the presence of DR. In patients with DR, retinal vascular permeability increased due to injury of the vascular endothelial cells and perivascular cells, and AGEs were thought to play critical roles in such vascular damage [16]. Accordingly, an increased risk of DR indicated by high AGEs levels detected in this study, likely explained by damage to the vascular cells, resulted in microvasculopathy development. Our results from the multivariate analysis show that smokers had lower AGEs scores (Table 9); a similar finding was detected in our previous study [8]. Smoking generally has been considered a factor in AGEs formation [17]. Given that the smokers in this study had lower Veggie scores (Table 10), suppression of appetite and/or promotion of metabolism through nicotine consumption might be an explanation for the discrepancy [18].

The study did not find a difference in Veggie scores among the control, NDR, and DR groups, whereas female sex, nonsmoking status, and better BCVA were associated with higher Veggie scores (Table 10). Higher carotenoid levels in women and nonsmoking status have been reported previously [19,20,21]. Higher dietary intake of carotenoids in women than men has also been reported [22]; thus, the current results are consistent with previous studies. The Veggie score clearly reflected the vegetable intake score (Table 8). Collectively, the results suggest that the fingertip measurement appropriately estimated the carotenoid levels. Provitamin A carotenoids, which are converted into vitamin A, are essential for maintaining the photoreceptor cell function. Macular pigment xanthophylls, such as lutein and zeaxanthin, are the major protectants of the foveal region via their antiphotooxidative stress effects [19]. Filtering of the harmful shorter wavelength blue light and/or elimination of singlet oxygen species explains the cellular protection activities of these macular pigment carotenoids [19]. Carotenoids were associated with better visual function by maintaining lens transparency [23] and macular function [24]. Considering these previous findings, the relationship between better BCVA and Veggie scores detected in this study (Table 5 and Table 10) seems reasonable. Contrary to expectations, higher Veggie scores were associated with higher DR risk (Table 3). More aggressive or rigorous dietary guidance applied to patients with DM/DR compared with controls might explain this result, although this needs to be tested.

We detected negative correlations between AGEs scores and Veggie scores (Table 4, Table 5, Table 9 and Table 10, Figure 1). AGEs accumulate in the body via two pathways, that is, in vivo formation through glycation reaction and dietary intake of AGEs, whereas all carotenoids are derived from dietary intake. Regarding the dietary intake of AGEs, subjects who preferred a “healthy” lifestyle likely exhibited a higher vegetable intake and lower AGEs foods; thus, this preference might explain the negative correlation between AGEs and carotenoid levels. Ingested carotenoids may inhibit AGEs synthesis, but the associated mechanisms should be studied further. Given that the AGEs and vegetable intake scores are inversely associated, and that higher AGEs levels were associated with the presence of DR, intensification of vegetable intake to prevent DR via inhibition of AGEs accumulation is a clinical consideration that arose from our results. This should be tested in future clinical studies.

The current study had several limitations. The retrospective nature of the study may have introduced biases. In multivariate analysis, the DR risk was higher in younger patients. Type I DM and young onset are generally associated with severe DM/DR; therefore, lack of information about the DM type (i.e., type I or II, duration, and severity of DM) is a study limitation. Because of the absence of a pre-estimated sample size, the absence of a significant difference in AGEs levels between controls and NDR patients in this study suggests either an actual absence of a difference, or a false absence of a difference due to weak detection power. Despite the limitations, we included all patients who satisfied the inclusion and exclusion criteria, thus limiting the selection bias.

## 5. Conclusions

The current results suggest that AGEs may have independent roles in DM/DR, and the AGEs score could be used as the indicator of these conditions. Although the roles of the carotenoid level in DM/DR were not determined, the Veggie score reflected the daily intake of vegetables, and was correlated inversely with the AGEs score. Skin sensors can be used to estimate the AGEs and carotenoid levels in clinical settings.

## Figures and Tables

**Table 1:** Demographic subject data.

Parameter	Control	DM	*p*-Value ^a^		NDR	DR	*p*-Value ^b^
N	156	93			49	44	
Age (years)							
Mean ± SD	69.9 ± 12.1	69.9 ± 12.0	1.00		74.2 ± 7.7	65.1 ± 14.0	0.0011 **
Range	38–92	31–90			53–90	31–88	
				*p*-value vs. control ^c^	0.0192	0.0254	
				*p*-value vs. NDR ^c^	-	0.0002 ^##^	
Sex							
Men, n (%)	69 (44.2)	61 (65.6)	0.0016 **		33 (67.4)	28 (63.6)	0.0048 **
Women, n (%)	87 (55.8)	32 (34.4)			16 (32.7)	16 (36.4)	
				*p*-value vs. control ^c^	0.0054 ^$$^	0.027 ^$^	
				*p*-value vs. NDR ^c^	-	0.83	
Mean blood pressure (mmHg)							
Mean ± SD	100.4 ± 15.3	99.2 ± 16.4	0.58		97.3 ± 16.2	101.3 ± 16.5	0.42
Range	68.3–142.3	47.3–156.3			47.3–138.0	70.7–156.3	
PR (cpm)							
Mean ± SD	77.5 ± 15.3	78.8 ± 11.7	0.51		76.8 ± 13.1	80.9 ± 9.8	0.33
Range	50.0–140.0	55.0–110.0			55.0–110.0	66.0–109.0	
BMI (kg/m^2^)							
Mean ± SD	22.4 ± 3.4	24.2 ± 4.48	0.0005 **		24.4 ± 4.9	24.0 ± 4.0	0.0022 **
Range	16.0–32.8	16.2–42.8			16.2–42.8	17.0–37.3	
				*p*-value vs. control ^c^	0.0022 ^##^	0.0090 ^#^	
				*p*-value vs. NDR ^c^	-	0.7100	
Current smoking status							
No, n (%)	139 (89.1)	78 (83.9)	0.25		39 (79.6)	39 (88.6)	0.22
Yes, n (%)	17 (10.9)	15 (16.1)			10 (20.4)	5 (11.4)	
Lens status							
Phakia	143 (91.7)	69 (74.2)	0.0003 **		42 (85.7)	27 (61.4)	<0.0001 **
Pseudophakia	13 (8.3)	24 (25.8)			7 (14.3)	17 (38.6)	
				*p*-value vs. control ^c^	0.2684	<0.0001 ^$$^	
				*p*-value vs. NDR ^c^	-	0.0093 ^$$^	
BCVA (logMAR)							
Mean ± SD	0.11 ± 0.21	0.49 ± 0.78	<0.0001 **		0.43 ± 0.62	0.57 ± 0.93	<0.0001 **
range	−0.08–1.00	−0.08–2.89			−0.08–2.70	−0.08–2.89	
				*p*-value vs. control ^c^	<0.0001 ^##^	<0.0001 ^##^	
				*p*-value vs. NDR ^c^	-	0.40	
IOP (mmHg)							
Mean ± SD	14.6 ± 2.8	15.4 ± 8.2	0.32		15.2 ± 3.1	15.6 ± 11.5	0.57
range	8.0–24.3	8.0–80.0			10.0–22.0	8.0–80.0	
AGEs scores (A.U.)							
Mean ± SD	0.44 ± 0.07	0.47 ± 0.09	0.029 *		0.45 ± 0.09	0.49 ± 0.08	0.0033 **
range	0.25–0.61	0.24–0.75			0.24–0.69	0.33–0.75	
				*p*-value vs. control ^c^	0.83	0.0006 ^##^	
				*p*-value vs. NDR ^c^	-	0.019	
Veggie scores (O.D.)							
Mean ± SD	327.7 ± 137.0	324.7 ± 126.4	0.86		320.4 ± 123.6	329.4 ± 130.8	0.93
range	123.5–812.0	63.5–719.0			63.5–719.0	111.0–547.0	
Vegetable intake scores							
0	9 (5.9)	3 (3.2)	0.46		2 (4.1)	1 (2.3)	0.70
1	27 (17.8)	23 (24.7)			13 (26.5)	10 (22.7)	
2	80 (52.6)	49 (52.7)			23 (46.9)	26 (59.1)	
3	36 (23.7)	18 (19.4)			11 (22.5)	7 (15.9)	

^a^ Comparison between control and DM groups by unpaired *t*-test or Fisher’s exact probability test. ^b^ Comparison among control, NDR, and DR groups by one-way ANOVA or G-test. ^c^ Comparison between control, NDR, and DR groups by post hoc unpaired *t*-test or Fisher’s exact probability test. Significance levels at 5% (*p* < 0.05) *, 1% (*p* < 0.01) **, 5% (*p* < 0.0167) ^#^, 1% (*p* < 0.0033) ^##^, 5% (*p* < 0.0167) ^$^, and 1% (*p* < 0.0033) ^$$^. PR, pulse rate; cpm, count per minute; BMI, body mass index; BCVA, best-corrected visual acuity; IOP, intraocular pressure.

**Table 2:** Multivariate logistic regression analysis for risk factors for DM.

Parameter	Unit OR	95% CI	*p*-Value ^a^
Entire model	-	-	<0.0001 **
Age (years)	0.99	0.96–1.03	0.68
Women (/men)	0.22	0.10–0.50	0.0001 **
Mean blood pressure (mmHg)	0.98	0.96–1.01	0.16
PR (cpm)	1.01	0.99–1.04	0.37
BMI (kg/m^2^)	1.12	1.02–1.23	0.018 *
Current smoking status, yes (/no)	2.85	0.96–8.40	0.057
Pseudophakia (/phakia)	6.18	2.08–18.3	0.0008 **
BCVA (logMAR)	22.6	4.31–118	<0.0001 **
IOP (mmHg)	1.04	0.912–1.18	0.59
AGEs score (A.U.)	122.5	0.71–21,272	0.059
Veggie score (O.D.)	1.00	1.00–1.01	0.11

^a^*p*-Values were calculated by the likelihood ratio test. Significance levels at 5% (*p* < 0.05) * and 1% (*p* < 0.01) **. Unit OR, odds ratio per unit of each parameter; CI, confidence interval; PR, pulse rate; cpm, count per minute; BMI, body mass index; BCVA, best-corrected visual acuity; IOP, intraocular pressure.

**Table 3:** Multivariate logistic regression analysis for risk factors for DR.

Parameter	Unit OR	95% CI	*p*-Value ^a^
Entire model	-	-	<0.0001 **
Age (years)	0.94	0.90–0.97	0.0006 **
Women (/men)	0.31	0.12–0.81	0.013 *
Mean blood pressure (mmHg)	1.01	0.98–1.05	0.37
PR (cpm)	1.02	0.99–1.06	0.2
BMI (kg/m^2^)	1.02	0.92–1.14	0.66
Current smoking status, yes (/no)	0.83	0.21–3.31	0.79
Pseudophakia (/phakia)	5.25	1.68–16.4	0.0047 **
BCVA (logMAR)	2.61	1.17–5.83	0.016 *
IOP (mmHg)	0.98	0.92–1.06	0.66
AGEs score (A.U.)	27,659	29.3–	0.0016 **
Veggie score (O.D.)	1.00	1.00–1.01	0.040 *

^a^*p*-Values were calculated using the likelihood ratio test. Significance levels at 5% (*p* < 0.05) * and 1% (*p* < 0.01) **. Unit OR, odds ratio per unit of each parameter; CI, confidence interval; PR, pulse rate; cpm, count per minute; BMI, body mass index; BCVA, best-corrected visual acuity; IOP, intraocular pressure.

**Table 4:** Possible associations between the AGEs score (A.U.) and various continuous parameters.

Parameter	*r*	Lower 95% CI	Upper 95% CI	*p*-Value
Age (years)	0.05	−0.08	0.17	0.47
Mean blood pressure (mmHg)	−0.08	−0.20	0.06	0.26
PR (cpm)	−0.07	−0.20	0.06	0.29
BMI (kg/m^2^)	0.06	−0.06	0.19	0.33
BCVA (logMAR)	0.12	−0.01	−0.24	0.065
IOP (mmHg)	−0.12	−0.25	0.01	0.068
Veggie score (O.D.)	−0.21	−0.33	−0.09	0.0007 **

The correlation coefficient (*r*) by Pearson’s correlation coefficient. ** Significance level at 1% (*p* < 0.01). CI, confidence interval; PR, pulse rate; cpm, count per minute; BMI, body mass index; BCVA, best-corrected visual acuity; IOP, intraocular pressure.

**Table 5:** Possible associations between the Veggie score (O.D.) and various continuous parameters.

Parameter	*r*	Lower 95% CI	Upper 95% CI	*p*-Value
Age (years)	0.13	0.00	0.25	0.049 *
Mean blood pressure (mmHg)	−0.02	−0.15	0.12	0.83
PR (cpm)	0.04	−0.10	0.17	0.59
BMI (kg/m^2^)	−0.09	−0.21	0.04	0.17
BCVA (logMAR)	−0.15	−0.27	−0.02	0.020 *
IOP (mmHg)	0.06	−0.07	0.19	0.38
AGEs score (A.U.)	−0.21	−0.33	−0.09	0.0007 **

The correlation coefficient (*r*) by Pearson’s correlation coefficient. Significance levels at 5% (*p* < 0.05) * and 1% (*p* < 0.01) **. CI, confidence interval; PR, pulse rate; cpm, count per minute; BMI, body mass index; BCVA, best-corrected visual acuity; IOP, intraocular pressure.

**Table 6:** Possible association among AGEs score (A.U.) and various categorical parameters.

Parameter	Mean ± SD (95% CI)	Mean ± SD (95% CI)	*p*-Value
Sex	Men, 0.46 ± 0.08 (0.45–0.47)	Women, 0.45 ± 0.08 (0.43–0.46)	0.21
Current smoking status	No, 0.46 ± 0.08 (0.45–0.47)	Yes, 0.41 ± 0.08 (0.38–0.44)	0.0010 **
Lens status	Phakic, 0.45 ± 0.08 (0.44–0.46)	Pseudophakic, 0.46 ± 0.10 (0.43–0.49)	0.42

*p*-Values calculated by *t*-test. ** Significance levels at 1% (*p* < 0.01). CI, confidence interval.

**Table 7:** Possible association among Veggie score (O.D.) and various categorical parameters.

Parameters	Mean ± SD (95% CI)	Mean ± SD (95% CI)	*p*-Value
Sex	Men, 302 ± 123 (280–323)	Women, 354 ± 139 (329–379)	0.0018 **
Current smoking status	No, 336 ± 133 (318–354)	Yes, 262 ± 115 (220–303)	0.0029 **
Lens status	Phakic, 318 ± 128 (301–335)	Pseudophakic, 376 ± 152 (325–427)	0.014 *

*p*-Values calculated by *t*-test. Significance levels at 5% (*p* < 0.05) * and 1% (*p* < 0.01) **.

**Table 8:** Possible associations of vegetable intake scores with AGEs scores (A.U.) and Veggie scores (O.D.).

Vegetable Intake Score	0	1	2	3	
N	12	50	129	54	
Parameter	Mean ± SD (95% CI)	Mean ± SD (95% CI)	Mean ± SD (95% CI)	Mean ± SD (95% CI)	*p*-Value ^a^
AGEs score (A.U.)	0.49 ± 0.06 (0.45–0.53)	0.44 ± 0.09 (0.42–0.47)	0.45 ± 0.08 (0.44–0.46)	0.46 ± 0.07 (0.44–0.48)	0.29
Veggie score (O.D.)	288 ± 92 (230–346)	273 ± 113 (241–306)	325 ± 129 (303–348)	396 ±141 (357–434)	<0.0001 **
	-	*p*-value ^b^ vs. 0, *p* = 0.6812	*p*-value ^b^ vs. 0, *p* = 0.3308	*p*-value ^b^ vs. 0, *p* = 0.0138 ^#^	
	-	-	*p*-value ^b^ vs. 1, *p* = 0.0137 ^#^	*p*-value ^b^ vs. 1, *p* < 0.0001 ^##^	
	-	-	-	*p*-value ^b^ vs. 2, *p* = 0.0012 ^##^	

^a^ Comparison among 4 vegetable intake score groups by one-way ANOVA. ^b^ Comparison between vegetable intake score groups by post hoc unpaired *t*-test. Significance levels at 1% (*p* < 0.01) **, 5% (*p* < 0.0083) ^#^, and 1% (*p* < 0.0016) ^##^. CI, confidence interval.

**Table 9:** Possible associations among AGEs scores (A.U.) and various parameters analyzed by multiple regression model.

Parameter	*r*	Lower 95% CI	Upper 95% CI	*p*-Value	Standard β
Entire model	-	-	-	<0.0001 **	-
Age (years)	0.001	0.000	0.002	0.14	0.11
Women (/men)	0.005	−0.006	0.016	0.41	0.06
Mean blood pressure (mmHg)	−0.000	−0.001	0.000	0.47	−0.05
PR (cpm)	−0.000	−0.001	0.000	0.47	−0.05
BMI (kg/m^2^)	0.000	−0.002	0.003	0.86	0.01
Current smoking status, yes (/no)	−0.028	−0.044	−0.012	0.0006 **	−0.26
Pseudophakia (/phakia)	−0.000	−0.016	0.016	0.99	0.00
BCVA (logMAR)	−0.002	−0.027	0.023	0.88	−0.01
IOP (mmHg)	−0.002	−0.004	0.000	0.07	−0.14
NDR (/control)	−0.014	−0.034	0.005	0.15	−0.15
DR (/control)	0.030	0.011	0.049	0.0025 **	0.31
Veggie score (O.D.)	−0.000	0.000	0.000	0.0001 **	−0.29

*p*-Values are calculated by a multiple regression model. Significance levels at 1% (*p* < 0.01) **. CI, confidence interval; PR, pulse rate; cpm, count per minute; BMI, body mass index; BCVA, best-corrected visual acuity; IOP, intraocular pressure.

**Table 10:** Possible associations among Veggie scores (O.D.) and various parameters analyzed by multiple regression model.

Parameter	*r*	Lower 95% CI	Upper 95% CI	*p*-Value	Standard β
Entire model	-	-	-	<0.0001 **	-
Age (years)		−0.1	3.1	0.07	0.13
Women (/men)	21.3	2.9	39.7	0.023 *	0.16
Mean blood pressure (mmHg)	−1.1	−2.3	0.1	0.08	−0.13
PR (cpm)	0.4	−0.9	1.7	0.54	0.04
BMI (kg/m^2^)	−1.7	−6.3	2.8	0.45	−0.05
Current smoking status, yes (/no)	−47.3	−74.3	−20.3	0.0007 **	−0.25
Pseudophakic (/phakic)	22.8	−3.6	49.3	0.09	0.12
BCVA (logMAR)	−46.5	−87.6	−5.3	0.027 *	−0.16
IOP (mmHg)	2.1	−1.4	5.5	0.24	0.09
NDR (/control)	−10.2	−43.3	22.8	0.54	−0.06
DR (/control)	31.9	−1.5	65.3	0.06	0.19
AGEs score (A.U.)	−479.5	−717.4	−241.6	0.0001 **	−0.28

*p*-Values are calculated by a multiple regression model. Significance levels at 5% (*p* < 0.05) * and 1% (*p* < 0.01) **. CI, confidence interval; PR, pulse rate: cpm, count per minute; BMI, body mass index; BCVA, best-corrected visual acuity; IOP, intraocular pressure.

## Data Availability

Data is contained within the article and Supplementary Materials.

## Supplementary Materials

The following supporting information can be downloaded at: https://www.mdpi.com/article/10.3390/antiox11071370/s1. Table S1: dataset underlying this manuscript.

## References

[1] Li J., Cao Y., Liu W., Wang Q., Qian Y., Lu P. (2019). Correlations among Diabetic Microvascular Complications: A Systematic Review and Meta-analysis. Sci. Rep..

[2] Morrison F., Shubina M., Turchin A. (2011). Encounter frequency and serum glucose level, blood pressure, and cholesterol level control in patients with diabetes mellitus. Arch. Intern. Med..

[3] Jampol L.M., Glassman A.R., Sun J. (2020). Evaluation and Care of Patients with Diabetic Retinopathy. N. Engl. J. Med..

[4] Stitt A.W., Curtis T.M., Chen M., Medina R.J., McKay G.J., Jenkins A., Gardiner T.A., Lyons T.J., Hammes H.P., Simo R. (2016). The progress in understanding and treatment of diabetic retinopathy. Prog. Retin. Eye Res..

[5] Bejarano E., Taylor A. (2019). Too sweet: Problems of protein glycation in the eye. Exp. Eye Res..

[6] Nowotny K., Jung T., Hohn A., Weber D., Grune T. (2015). Advanced glycation end products and oxidative stress in type 2 diabetes mellitus. Biomolecules.

[7] Takayanagi Y., Yamanaka M., Fujihara J., Matsuoka Y., Gohto Y., Obana A., Tanito M. (2020). Evaluation of Relevance between Advanced Glycation End Products and Diabetic Retinopathy Stages Using Skin Autofluorescence. Antioxidants.

[8] Shirakami T., Yamanaka M., Fujihara J., Matsuoka Y., Gohto Y., Obana A., Tanito M. (2020). Advanced Glycation End Product Accumulation in Subjects with Open-Angle Glaucoma with and without Exfoliation. Antioxidants.

[9] Kadoh Y., Takayanagi Y., Sasaki J., Tanito M. (2022). Fingertip-Measured Skin Carotenoids and Advanced Glycation End Product Levels in Glaucoma. Antioxidants.

[10] Zheng J.S., Sharp S.J., Imamura F., Chowdhury R., Gundersen T.E., Steur M., Sluijs I., van der Schouw Y.T., Agudo A., Aune D. (2020). Association of plasma biomarkers of fruit and vegetable intake with incident type 2 diabetes: EPIC-InterAct case-cohort study in eight European countries. BMJ.

[11] Sugiura M., Nakamura M., Ogawa K., Ikoma Y., Yano M. (2015). High-serum carotenoids associated with lower risk for developing type 2 diabetes among Japanese subjects: Mikkabi cohort study. BMJ Open Diabetes Res. Care.

[12] Meerwaldt R., Graaff R., Oomen P.H.N., Links T.P., Jager J.J., Alderson N.L., Thorpe S.R., Baynes J.W., Gans R.O.B., Smit A.J. (2004). Simple non-invasive assessment of advanced glycation endproduct accumulation. Diabetologia.

[13] Yamanaka M., Matsumura T., Ohno R., Fujiwara Y., Shinagawa M., Sugawa H., Hatano K., Shirakawa J., Kinoshita H., Ito K. (2016). Non-invasive measurement of skin autofluorescence to evaluate diabetic complications. J. Clin. Biochem. Nutr..

[14] Ermakov I.V., Ermakova M., Sharifzadeh M., Gorusupudi A., Farnsworth K., Bernstein P.S., Stookey J., Evans J., Arana T., Tao-Lew L. (2018). Optical assessment of skin carotenoid status as a biomarker of vegetable and fruit intake. Arch. Biochem. Biophys..

[15] Koetsier M., Nur E., Chunmao H., Lutgers H.L., Links T.P., Smit A.J., Rakhorst G., Graaff R. (2010). Skin color independent assessment of aging using skin autofluorescence. Opt. Express.

[16] Xie Y., Yu D., Wu J., Li L. (2017). Protective effects of physiological testosterone on advanced glycation end productinduced injury in human endothelial cells. Mol. Med. Rep..

[17] Nicholl I.D., Bucala R. (1998). Advanced glycation endproducts and cigarette smoking. Cell Mol. Biol..

[18] Thorgeirsson T.E., Gudbjartsson D.F., Sulem P., Besenbacher S., Styrkarsdottir U., Thorleifsson G., Walters G.B., Furberg H., Sullivan P.F., Marchini J. (2013). A common biological basis of obesity and nicotine addiction. Transl. Psychiatry.

[19] Takayanagi Y., Obana A., Muto S., Asaoka R., Tanito M., Ermakov I.V., Bernstein P.S., Gellermann W. (2021). Relationships between Skin Carotenoid Levels and Metabolic Syndrome. Antioxidants.

[20] Matsumoto M., Suganuma H., Shimizu S., Hayashi H., Sawada K., Tokuda I., Ihara K., Nakaji S. (2020). Skin Carotenoid Level as an Alternative Marker of Serum Total Carotenoid Concentration and Vegetable Intake Correlates with Biomarkers of Circulatory Diseases and Metabolic Syndrome. Nutrients.

[21] Sugiura M., Nakamura M., Ogawa K., Ikoma Y., Matsumoto H., Ando F., Shimokata H., Yano M. (2008). Associations of serum carotenoid concentrations with the metabolic syndrome: Interaction with smoking. Br. J. Nutr..

[22] Bohm V., Lietz G., Olmedilla-Alonso B., Phelan D., Reboul E., Banati D., Borel P., Corte-Real J., de Lera A.R., Desmarchelier C. (2021). From carotenoid intake to carotenoid blood and tissue concentrations-implications for dietary intake recommendations. Nutr. Rev..

[23] Cui Y.H., Jing C.X., Pan H.W. (2013). Association of blood antioxidants and vitamins with risk of age-related cataract: A meta-analysis of observational studies. Am. J. Clin. Nutr..

[24] Feng L., Nie K., Jiang H., Fan W. (2019). Effects of lutein supplementation in age-related macular degeneration. PLoS ONE.

