# Supplementary material for: Relevance of Diabetic Retinopathy with AGEs and Carotenoid Levels Assessed by Skin Sensors
Source: Antioxidants (Basel). 2022 Jul 14;11(7):1370. doi: 10.3390/antiox11071370 (PMC9311940; doi:10.3390/antiox11071370)
Supplement: Supplementary file 1 [file antioxidants-11-01370-s001.zip › antioxidants-1790616-supplementary.pdf]

Study ID	Group	Age	sex	AGEs	Veggie	Smoke	BMI	BP	PR	Vegetable	Lens	BCVA	IOP
1	control	72	F	0.455	224	0	23.24459	99.33333	65		phakia	0.09691	16
3	control	62	M	0.445	256	0	23.52941				IOL	-0.07918	11
31	NDR	76	F	0.24	409.5	1	19.99635	100.6667	76	2	phakia	0.39794	20
38	NDR	74	F	0.36	434	0	28			2	phakia	0.154902	14
41	control	71	M	0.425	564	1	19.53125			3	phakia	0.39794	13
48	control	45	M	0.475	178	1	20.30517	96	86	2	phakia	-0.07918	11
49	control	84	F	0.595	254	0	27.4974	106.3333	59	2	phakia	0.221849	15
51	control	38	M	0.43	547	0		80		2	phakia	-0.07918	19
52	NDR	85	F	0.41	325.6667	0		105	78	2	phakia	0.39794	14.3
53	control	88	F	0.35	294.5	0	16.82023			2	IOL	-0.07918	10
56	control	61	F	0.295	657	0	19.36913	90.66667	63	2	phakia	-0.07918	
57	control	88	F	0.485	587	0	21.14316	83.66667	68	3	phakia	0.39794	10.3
58	DR	76	M	0.745	445	0	24.84987	89.66667	93	3	IOL	-0.07918	10
60	control	70	M	0.475	548	0	21.62434	88.66667	66	3	phakia	0.221849	19
67	DR	72	M	0.465	319.5	0	23.59151	92	70	2	phakia	0.221849	9
69	control	78	F	0.435	354.5	0	23.72529			0	phakia	0.30103	13
75	control	61	F	0.445	390.5	0	19.8791	90	76	2	phakia	0.09691	12
78	control	79	F	0.415	382	0	21.1109	100	80	3	IOL	-0.07918	13
79	control	73	F	0.405	481.5	0	21.33333	94	72	2	IOL	-0.07918	15
93	control	63	M	0.46	375	0		97.33333	95	2	phakia	0	19
94	control	62	F	0.525	225.5	0	24.65303	132.6667	107	0	phakia	-0.07918	18
103	control	48	F	0.585	226.5	0	22.1872			1	phakia	-0.07918	16
121	control	68	M	0.415	202.5	1	18.55677	68.33333	108	0	phakia	0.522879	15
122	control	48	F	0.46	164.5	1	19.47341	97.66667	69	0	phakia	-0.07918	17
123	control	68	F	0.315	461.5	0	19.81784	139	140	2	IOL	-0.07918	17
125	control	67	F	0.365	704	0	21.92886	83.66667	82	2	phakia	0.045757	14
132	control	88	F	0.53	306	0	17.06011			2	phakia	-0.07918	14
140	control	80	F	0.52	468	0	22.77563		79	3	phakia	0.30103	16
151	NDR	72	F	0.48	411.5	0	18.66201			3	phakia	0.221849	14
154	DR	64	F	0.565	471.5	0	25.07812	86	87	3	IOL	0.823909	9
157	control	75	F	0.465	252.5	0	24.40105	105.6667	91	2	phakia	0.30103	17
159	control	88	F	0.52	569	0	24.4352			3	phakia	0.39794	18
160	control	68	F	0.455	457.5	0	22.80946	97.66667	72	2	phakia	0.221849	11
171	DR	88	M	0.455	507.5	0	24.18175	95	76	3	IOL	0	10
175	NDR	74	M	0.485	353	1		104.6667	66	2	phakia	0	19
177	control	73	F	0.45	534.5	0	21.65876	82.66667	64	2	IOL	-0.07918	18
178	control	70	M	0.55	260.5	0	24.28283			0	phakia	0	18
188	control	81	M	0.605	262.5	0		100	95	2	phakia	0	
190	control	61	F	0.45	424	0		88.33333	79	3	phakia	0	
191	NDR	76	M	0.57	256.5	0	21.63332	77.33333	73	1	IOL	-0.07918	14
207	DR	72	M	0.505	347.5	0		134	87	1	IOL	0.39794	16
218	NDR	74	M	0.5	413	0	24.4646			2	phakia	0.69897	14
219	control	80	M	0.48	258.5	0	20.70313			2	phakia	0.045757	14
235	control	72	F	0.41	297.5	0	23.89779			2	phakia	0	
249	NDR	78	M	0.38	303	1	31.43859	89.66667	87	3	phakia	0.154902	16
287	control	82	F	0.395	463.5	0	22.22222	138.6667	104	3	IOL	0	
300	control	72	F	0.555	409.5	0	15.97882	92	81	0	phakia	0.154902	12
325	DR	41	F	0.555	438.5	0	19.14672	110	96	0	IOL	0.221849	19
327	control	67	F	0.3	480	0	19.31295	103.6667	68	2	phakia	0.09691	18
328	control	83	F	0.5	248.5	0	19.97622	103.6667	68	2	phakia	0.154902	12
329	control	84	F	0.545	435.5	0	20.8292	109.6667	112	3	phakia	0.154902	17
337	DR	73	M	0.515	473	0	23.03005	106.3333	81	2	phakia	0.39794	11
350	NDR	80	M	0.455	417	0	25.65437	84.66667	58	3	phakia	0.30103	11
370	control	71	M	0.51	228.5	0	21.72547	105	86	1	phakia	0.221849	15
371	DR	77	M	0.42	542	0	22.64086	119.3333	81	2	IOL	0.221849	9.3
398	control	78	M	0.44	375	1	17.31341	88.33333	83	2	phakia	0.154902	16
399	control	71	F	0.515	203.5	0	20.73688	116	82	1	phakia	0.823909	15
401	control	64	M	0.565	129.5	0	24.07407	127.3333	61	2	phakia	-0.07918	13
402	NDR	80	M	0.41	719	0	24.83576	86	75	3	IOL	0	22
408	control	70	F	0.595	230.5	0	22.26563	99	98	1	phakia	-0.07918	14
410	DR	42	F	0.56	342	1	37.32403	106.6667	88	1	phakia	-0.07918	25
413	DR	67	F	0.645	140.5	0	23.30905	70.66667	82	2	phakia	2.69897	13
416	control	82	F	0.55	423.5	0	22.4323	82	79	3	phakia	0	18
421	control	77	F	0.555	210.5	0	27.63037	93	66	3	phakia	0.045757	14.7
434	NDR	76	M	0.42	376.5	0	22.40588	83.66667	95	1	phakia	0	15
436	DR	49	M	0.405	412.5	0	28.87544	89	76	2	phakia	0.30103	17
459	DR	64	M	0.73	304	0	24.33748	108.6667	79	2	IOL	0.09691	
475	control	63	F	0.505	434.5	0	26.05835	96	69	2	phakia	-0.07918	
486	control	72	M	0.475	328	0	21.03211	116	89	2	phakia	-0.07918	17
494	control	75	M	0.545	202.5	0	19.47341	97.66667	78	2	phakia	0.823909	
504	DR	72	F	0.515	238.5	0	22.4323			2	IOL	1	
524	control	77	F	0.465	372.5	0	24.39482	117.6667	79	2	phakia	0.30103	
537	control	71	M	0.435	130.5	1	23.30668	87.66667	89	2	phakia	0.09691	
542	DR	75	F	0.465	446.5	0	25	106	66	2	phakia	-0.07918	13.7
574	DR	55	M	0.54	196.5	1	20.57613	96	69	2	IOL	2.79588	
576	NDR	82	F	0.49	418.5	0	21.70139	104	58	2	phakia	1.69897	
604	DR	67	M	0.42	470	0	21.51386	92	79	2	phakia	0	13.3
614	DR	64	M	0.405	411.5	0	18.72417	104.3333	92	2	IOL	2	8

623	NDR	79	M	0.44	215.5	0	21.45329	70.33333	68	2	IOL	-0.07918	10
657	control	81	M	0.425	408	0	16.20308	78	72	3	IOL	0.221849	10
658	control	87	F	0.505	172	0	25.05565	89	73	2	phakia	0.09691	11
660	DR	67	F	0.455	120	0	23.01118	75	93	2	IOL	2.60206	
675	DR	78	M	0.44	252	0	22.03173	93	79	2	phakia	0.045757	11.7
676	control	70	F	0.395	224.5	0	22.07108	91.33333	72	3	phakia	0.045757	
677	DR	61	F	0.59	111	0	29.756	87.33333	67	2	phakia	2	
683	control	78	F	0.4	258.5	0	32.83918	105	98	2	phakia	1	13
685	control	76	F	0.305	498.5	0	22.64086	126.3333	81	2	phakia	0	14
691	NDR	73	M	0.385	63.5	1	22.71897	96.66667	82	2	phakia	0.221849	15.3
714	control	63	M	0.515	286	0	25.68956	91.33333	54	3	phakia	-0.07918	16.3
725	NDR	65	M	0.635	169.5	0	27.4406	82.66667	79	3	phakia	0.39794	
726	NDR	66	M	0.41	235	0	33.42516	105.3333	69	2	phakia	2.60206	12
729	control	80	M	0.43	252.5	0	28.80441	113	50	1	phakia	0.154902	14
731	control	53	M	0.52	181.5	0	27.04164	72.33333	55	2	phakia	0	17.7
751	NDR	78	F	0.36	445	0	21.84586			3	phakia	0.30103	16
756	NDR	77	M	0.515	434	0	21.82995	106	55	2	phakia	0.154902	14
757	control	88	F	0.495	349.5	0	22.95918	110.6667	56	2	phakia	0.39794	16
767	DR	71	F	0.53	298	0	26.27135	101.3333	81	2	phakia	0.69897	10
769	control	81	F	0.335	308.5	0	23.92569	99.33333	73	2	IOL	0.30103	13.7
773	control	84	M	0.53	170	0	16.14153	114.3333	73	1	phakia	0.154902	
784	NDR	53	F	0.3	428	0	28.76397	91	66	2	phakia	0.154902	18
786	NDR	71	F	0.495	517	0	28.39872	99.66667	91	1	phakia	0.30103	14.7
797	control	77	F	0.46	437.5	0	24.11265	129.3333	69	3	phakia	-0.07918	18
803	control	47	F	0.31	349	1	22.67574	103.6667	86	1	phakia	-0.07918	17
804	control	55	F	0.55	267	0	29.64269	89.66667	67	3	phakia	0	15.3
805	control	81	M	0.34	414	0	22.82688	95	73	1	phakia	0.221849	16
806	control	59	M	0.5	244.5	0	22.85714	108.3333	67	2	phakia	0	17.7
808	NDR	83	M	0.6	220	0	24.97399	65.33333	70	1	phakia	2	
809	control	56	F	0.315	333.5	0	22.83288	99.33333	66	2	phakia	-0.07918	24.3
810	control	70	F	0.41	237.5	0	27.82931	109.3333	74	2	phakia	0.154902	13
811	control	43	M	0.5	255.5	0	27.68166	84.33333	54	3	phakia	0.09691	12
812	control	69	M	0.42	320.5	0	28.28283	93.66667	70	2	phakia	0.154902	19
825	control	42	M	0.385	371.5	1	26.98962	88.66667	58	0	phakia	-0.07918	18
837	NDR	67	M	0.465	260	0	27.96802	110	84	0	IOL	-0.07918	
840	control	54	M	0.445	279.5	0	25.0995	86.66667	65	2	phakia	-0.07918	14.7
843	control	92	F	0.385	296.5	0	23.55556	101	88	3	phakia	0.69897	14
857	control	61	M	0.45	227	0	24.6181	79	67	2	phakia	-0.07918	13.3
861	control	73	F	0.3	139.5	0	17.51463	85.33333	77	1	phakia	0.045757	
863	control	61	F	0.365	233.5	0	19.83471	75.66667	69	2	phakia	0.522879	12
869	NDR	75	M	0.43	255.5	0	16.18427	87	56	3	phakia	0.30103	13
871	control	80	F	0.46	547	0	19.02497	81.66667	60	3	phakia	0	8
877	control	75	M	0.415	450	0	19.53125	88.33333	65	3	phakia	0.045757	13.7
879	NDR	81	F	0.455	293	0	22.52151	83.66667	103	2	phakia	0.522879	12
880	DR	72	M	0.46	547	0	27.34375	97	68	2	phakia	0	11
882	NDR	58	M	0.445	231.5	1	32.8473	117.3333	82	1	phakia	0.69897	19
883	NDR	69	M	0.27	182	1	16.40625	47.33333	62	1	phakia	0.30103	18
885	control	74	F	0.435	175.5	0	19.73598	85	69	0	phakia	0.39794	10.3
886	control	70	M	0.555	128.5	0	23.53304			1	phakia	-0.07918	17
888	control	43	M	0.265	233	1	17.99015	133.6667	108	1	phakia	-0.07918	14
898	NDR	69	M	0.545	205	0	22.40588	118.6667	60	2	phakia	0	14
912	control	62	M	0.415	274.5	0	19.72318	122.3333	104	3	phakia	-0.07918	19.3
913	control	60	F	0.375	173	0	17.2248	142.3333	65	1	phakia	0.39794	20
917	NDR	71	F	0.43	479.5	1	20.8292	110.3333	83	2	phakia	0	16
918	control	75	M	0.32	273	1	25.10239	122.6667	91	2	phakia	-0.07918	16
921	NDR	86	F	0.685	222.5	0	17.77778	98.33333	74	2	phakia	1	16
923	control	71	F	0.365	315	0	20.93664	105	73	2	phakia	0.154902	17
934	control	78	F	0.25	257.5	0	19.42869				phakia	0	15
935	control	71	F	0.475	608	0	20.54419	99	72	3	phakia	-0.07918	18
937	NDR	78	M	0.395	506.5	0	24.88281	105	69	3	phakia	0.39794	15
939	NDR	77	F	0.475	351.5	0	28.88889	81	77	1	IOL	0.69897	11.3
941	NDR	90	M	0.515	305	0	19.92513	138	84	2	phakia	0.823909	13.3
943	control	59	M	0.415	140.5	0	21.45329				phakia	0.221849	8
947	control	80	F	0.5	198	0	22.86237	96.33333	65	3	phakia	0.154902	14
949	DR	84	M	0.42	314	0	20.76125	115.6667	79	2	phakia	0.09691	14
950	DR	68	M	0.48	195.5	0	22.03857	100	77	2	phakia	-0.07918	13.7
954	control	68	M	0.35	247	0	25.43269	99	52	2	phakia	0.154902	14
964	control	80	M	0.49	401.5	0	23.73866	114.3333	97	2	phakia	0.221849	10
966	control	58	F	0.45	156.5	0	26.31464	119	69	1	phakia	-0.07918	17.3
967	control	75	M	0.53	333	0	18.35938	108.3333	63	0	phakia	0.221849	14
968	NDR	68	M	0.38	339.5	1	25.40282	98.33333	81	1	phakia	0.30103	10
969	control	85	F	0.475	153.5	0	22.82688	81	85	2	phakia	0.39794	
977	control	74	F	0.455	371.5	0	21.35991	112.3333	91	3	phakia	0.221849	10
981	control	61	M	0.415	200.5	1	30.42185	118	56	3	phakia	-0.07918	18
984	DR	72	M	0.445	177.5	0	27.92667			2	phakia	2.69897	12
988	DR	31	F	0.535	148.5	0	26.95313			3	phakia	-0.07918	28
991	control	70	M	0.43	252.5	0	21.79931	109.6667	54	2	phakia	0.221849	10
992	control	83	M	0.53	306.5	0	21.2585	102.6667	66	2	phakia	0.30103	14
996	control	55	M	0.45	308	0	28.71048	102.6667	92	2	phakia	0.69897	16.7

997	control	82	M	0.425	246.5	0	19.59184	79	79	1	phakia	0.154902	
1004	control	81	M	0.49	269	0	18.68512			2	phakia	0.09691	17
1005	control	73	F	0.515	225	0	20.44444	83.33333	75	2	phakia	0.30103	20.3
1006	control	55	M	0.43	290.5	0	29.72652	112.6667	80	1	phakia	-0.07918	16
1009	control	67	F	0.45	450.5	0	18.76525	97	81	1	phakia	0.221849	13
1013	control	71	F	0.425	393	0	16.89189	109.3333	124	3	phakia	0	14
1017	control	67	F	0.475	293.5	0	19.97919	93.33333	77	3	phakia	0.30103	
1018	DR	65	M	0.465	262.5	0	16.90103	84.66667	75	1	IOL	-0.07918	11
1019	control	65	M	0.415	172	1	22.83737	118.6667	81	2	phakia	-0.07918	18
1025	NDR	70	M	0.435	212	0	29.37758	113	60	2	phakia	0.39794	20
1027	control	78	M	0.425	190.5	1	21.48438	104.3333	87	2	phakia	0.30103	12
1031	control	54	F	0.385	277.5	0	22.0741	76.33333	80	2	phakia	-0.07918	13
1039	control	85	M	0.515	139.5	0	23.30905	89	77	2	phakia	0.30103	11.7
1040	control	60	F	0.475	368.5	0	23.01118	100	71	2	phakia	-0.07918	14
1050	control	73	F	0.47	271.5	0	17.34843	80.33333	81	2	phakia	-0.07918	12
1052	NDR	82	M	0.46	372.5	0	22.18935	100	61	1	phakia	0.39794	13
1055	control	80	M	0.355	133.5	1	23.42209	102	74	2	phakia	0	16
1056	control	81	M	0.35	345	0	25.06575	100.6667	88	2	phakia	0.09691	14
1058	NDR	89	M	0.54	259	0	19.53125	114.6667	96	0	phakia	0.39794	13
1079	control	71	M	0.445	464.5	0	21.51386	104.3333	83	3	phakia	0.09691	12
1084	control	74	M	0.445	227.5	0	27.88519	94.66667	81	2	phakia	0	12
1085	NDR	74	M	0.435	217	1	25.81663	101	68	2	phakia	0.39794	16
1089	DR	61	M	0.48	372	0	22.58271	115.3333	69	2	phakia	0.09691	17
1090	DR	33	F	0.415	269	0	32.87071	95	76	2	phakia	-0.07918	19
1097	NDR	71	F	0.37	390.5	0	42.80618	116	88	2	phakia	0.823909	18
1100	DR	63	M	0.425	284	0	25.46939	105.6667	86	1	phakia	1.39794	17
1101	control	71	M	0.28	579.5	0	23.04688	83.66667	70	3	phakia	-0.07918	15
1109	control	79	M	0.46	513	0	21.08281	121.6667	69	3	IOL	-0.07918	12
1110	control	79	F	0.42	323	0	22.07108	103.3333	74	2	phakia	0	14
1115	control	73	M	0.39	185.5	0	20.40816	120.6667	112	1	IOL	-0.07918	14
1120	DR	58	F	0.446667	449.5	1	17.85062	120	98	2	IOL	-0.07918	17
1133	control	61	M	0.49	249	1	23.45856	109.6667	66	1	phakia	-0.07918	11.3
1134	DR	76	M	0.475	159	0	19.1953	119	70	3	phakia	0.30103	16
1135	NDR	75	M	0.555	294.5	0	21.56454	83.66667	95	2	phakia	0.30103	18
1137	NDR	71	F	0.41	428.5	0	19.73598	98.33333	84	2	phakia	0.39794	
1146	control	50	F	0.445	663	0	16.82423	75.33333	89	3	phakia	-0.07918	9.7
1149	control	79	F	0.405	551.5	0	16	108.3333	99	3	phakia	0.09691	16
1150	NDR	69	M	0.49	150	0	23.18339	108.6667	74	1	phakia	2.69897	15
1152	control	68	M	0.425	230	0	22.57105	83.66667	64	2	phakia	0	12
1153	DR	63	F	0.455	266.5	0	28.06122	96.33333	94	2	phakia	0.69897	13
1156	DR	73	F	0.43	503	0	19.56946	110	109	1	phakia	0.823909	13
1157	control	75	F	0.46	239	0	24.88889	104.3333	88	2	IOL	-0.07918	18
1158	control	38	M	0.425	298.6667	0	21.13886	81.66667	61	1	phakia	-0.07918	12
1164	DR	56	M	0.39	308	1	22.72044	111.3333	76	1	phakia	-0.07918	18
1168	control	42	M	0.395	295.5	0	21.7502	72.66667	71	2	phakia	-0.07918	16
1171	control	73	M	0.37	610.5	0	22.58955	91.66667	73	2	phakia	-0.07918	13.3
1172	control	71	F	0.485	483.5	0	19.81784	98	79	2	phakia	0	15
1178	control	82	F	0.495	537.5	0	24.19649	94.33333	56	2	phakia	0	20.3
1185	NDR	78	M	0.495	337	0	28.87544	100	80	2	IOL	0	16
1194	control	83	F	0.46	483	0	26.02264	107	63	2	phakia	0.221849	13.7
1197	NDR	79	M	0.37	427	0	21.00767	81.66667	94	3	phakia	0.045757	21
1198	control	43	F	0.455	309.5	0	27.68166	86.66667	61	2	phakia	0.154902	12
1207	control	60	F	0.35	268	0	21.23057	106.6667	79	1	phakia	0.09691	14
1212	control	44	F	0.47	302.6667	0	28.35306	90	70	1	phakia	0	10.7
1226	control	72	M	0.42	313.5	0	18.55677	105.3333	78	2	phakia	0.30103	14
1229	control	70	M	0.405	187	1	24.60938	107	82	2	phakia	0.045757	14
1230	NDR	85	M	0.49	229	0	23.87511	97.33333	83	2	IOL	0	14
1240	DR	74	F	0.33	467	0	27.81065	156.3333	91	1	IOL	2.886057	80
1245	control	66	F	0.375	490.6667	0	20.06095	119.6667	80	1	phakia	0.522879	15
1246	NDR	68	M	0.433333	282.5	0	23.95123	115.3333	67	3	phakia	0.045757	
1252	DR	51	M	0.52	262	0	28.08901	99.66667	82	2	phakia	-0.07918	10
1253	control	80	F	0.445	349.5	0	22.64086	97.66667	71	1	phakia	0.30103	15
1258	control	69	M	0.445	220.5	0	26.85441	85.66667	72	3	phakia	-0.07918	13.7
1259	NDR	62	F	0.4	400	0	22.05219	116	81	3	phakia	-0.07918	20
1263	control	72	F	0.345	414.5	0	29.33333	104.3333	94	2	phakia	0.30103	14
1264	DR	74	M	0.49	540.5	0	25.5102	109.3333	85	2	IOL	-0.07918	
1266	DR	81	M	0.495	317	0	22.30815	81.66667	68	3	IOL	-0.07918	11
1281	control	91	F	0.335	413.5	0	20.25463	113.6667	69	3	phakia	0.221849	
1288	DR	47	M	0.59	214.5	1	22.23099	76.66667	67	1	phakia	-0.07918	14
1291	control	48	F	0.495	157.6667	0	22.48133	93	73	1	phakia	-0.07918	11
1292	control	84	F	0.27	812	0	21.45727	84.33333	70	2	IOL	0.045757	14
1303	control	71	M	0.395	225.5	0	25.33333	119.3333	61	2	phakia	-0.07918	16
1310	control	74	M	0.495	324	0	24.21875	118.6667	77	2	phakia	0.09691	11
1318	control	81	F	0.37	562.5	0	20.54419	86	117	1	phakia	0.154902	12
1319	DR	85	F	0.43	154	0	19.2974	112.3333	85	1	phakia	0.09691	16
1321	NDR	57	M	0.295	139.5	1	26.72287	83.66667	90	1	phakia	-0.07918	11.3
1323	NDR	72	M	0.435	85	0	21.00767	103.3333	110	1	phakia	0.221849	
1324	DR	66	M	0.52	383.5	0	22.15102	75.33333	76	3	phakia	0.30103	12
1344	DR	82	M	0.52	138	0	22.94213	112.6667	81	1	IOL	-0.07918	10

1353	control	61	M	0.475	243	0	16.16162	100	81	2	phakia	-0.07918	16
1356	control	66	F	0.485	487.5	0	19.65056	87.33333	80	3	phakia	0	12
1361	control	42	F	0.55	220.5	0	26.43807	87.33333	94	1	phakia	-0.07918	17
1369	DR	34	M	0.36	474.5	0	26.89232	96.66667	82	2	phakia	0.154902	15.3
1372	control	65	M	0.395	167.5	1	19.05197	131.3333	91	1	phakia	0.30103	18
1373	control	74	M	0.52	443	0	26.17519	107.3333	92	2	phakia	0	14
1374	NDR	74	F	0.435	280	0	28.53746	96.33333	66	1	phakia	0.09691	10.3
1383	control	58	F	0.45	156.5	0	28.88889	110	74	2	phakia	-0.07918	17.3
1385	control	83	M	0.61	296	0	23.14726	118	61	2	phakia	0.39794	14
1391	control	73	F	0.405	326.5	0	17.44493	131	101	2	phakia	0.09691	17
1399	control	73	M	0.455	446.3333	0	24.22145	105.3333	74	2	phakia	-0.07918	17
1400	control	92	F	0.5	123.5	0	20.3428	106.6667	103	3	phakia	0.522879	14
1408	control	74	F	0.48	371	0	21.92886	91.33333	56	2	phakia	0.154902	10
